# ACORN: Adaptive Consensus of Repeated Clusterings Identifies Pyramidal Neurons and Interneurons in the Primate Motor Cortex Based on Extracellular Spike Waveforms

**DOI:** 10.1523/ENEURO.0394-25.2026

**Published:** 2026-10-23

**Authors:** Zoltan Soltesz, Viviane S. G. M. Tenorio, Takamichi Tohyama, Anne M. E. Baker, Stuart N. Baker, Alexander Kraskov

**Affiliations:** ^1^Faculty of Medical Sciences, Newcastle University, Newcastle NE2 4HH, United Kingdom; ^2^School of Engineering and Built Environment, Sheffield Hallam University, Sheffield S1 1WB, United Kingdom; ^3^Department of Rehabilitation Medicine, School of Medicine, Fujita Health University, Aichi 470-1192, Japan

**Keywords:** antidromic identification, electrophysiology, ground truth dataset, neuron classification, pyramidal tract neurons, supplementary motor area

## Abstract

Understanding how neural circuits generate behavior requires knowledge of both neural activity patterns and the cell types they originate from. While the scale of neuronal recordings has dramatically increased since the advent of dense multicontact probes, accurate identification of cell types remains elusive. Traditional approaches have used spike width to attempt a classification, with narrow spikes assumed to be inhibitory interneurons. However, fast pyramidal tract neurons (PTNs; known to be excitatory) with thin spikes and interneurons with wide spikes challenge these assumptions. Here we accomplish three related objectives. First, we produce a ground truth dataset of extracellular spike shapes from electrophysiologically identified pyramidal neurons from primary (M1) and supplementary motor areas (SMA) and optogenetically identified interneurons in M1 in female macaque monkeys. Next, we show that spike width correlates with antidromic latency not only in M1 but also in the SMA, suggesting that it is a physiologically meaningful measure across the motor cortex. Finally, we introduce our unsupervised ensemble clustering method, “ACORN,” and characterize its performance on our neocortical macaque dataset, the C4 cerebellar database, and a juxtacellularly recorded mouse dataset, demonstrating strong out-of-the-box performance across species, brain areas, and recording techniques. ACORN can cluster neuronal waveforms without training or tuning and provides data-derived confidence values for its predictions. Our ground truth data can be used as a reference to help identify or verify clusters in novel datasets. Together, our method and database provide a streamlined foundation for electrophysiological cell classification without necessitating changes to recording paradigms.

## Significance Statement

High-density extracellular recordings are increasingly the norm in neuroscience, but accurate and noninvasive identification of cells remains a challenge. Here we provide a database of action potential spike shapes from pyramidal tract neurons and interneurons of the macaque supplementary motor area, whose identities we verified using nerve stimulation and optogenetics, respectively. We introduce the neuron clustering method “ACORN” and showcase its robustness by evaluating its performance on a variety of distinct datasets. Our training-free clustering technique is designed to support experimentalists, while our ground truth database can enhance and validate future datasets and method development.

## Introduction

Different classes of neurons perform distinct roles within cortical networks. To understand how information is processed, knowledge of activity levels alone may not be sufficient; identifying cell types is a critical part of the puzzle. For example, detecting a high firing rate of local inhibitory interneurons would imply a different type of neural computation than the same pattern observed in excitatory pyramidal neurons that project to distant targets.

The prospect of reliably classifying cells based on their extracellular electrophysiological properties is appealing for several reasons. Firstly, using the same recordings to classify neurons and measure their activity patterns would make experiments more straightforward to perform. Additionally, it would allow building cell-specific computational models of complex animal behavior based on recordings in awake animals, without the need for additional, often postmortem, techniques required to confirm cell identity. Furthermore, it would facilitate more accurate targeting of cells of interest, by making it possible to adjust electrode position based on live information about cell types. Finally, it would enable real-time adjustments of electric, optogenetic, or pharmacological network manipulations in awake behaving animals as a function of observed cell type-specific activity.

Previous works suggested that neocortical interneurons have short action potentials, which could be used to distinguish them from pyramidal neurons that spike more rarely but with longer durations. Many subsequent studies have relied on action potential durations as a way to distinguish between putative interneurons and excitatory pyramidal cells (for an overview, see Table 2 in Vigneswaran et al., 2011). However, the considerable diversity of cortical interneurons, with multiple morphological subtypes clustering into the same electrophysiological classes (Zaitsev et al., 2009), raised questions about the reliability of such an approach.

The picture is further complicated by different brain regions having distinct compositions of cell types. For example, giant Betz cells are present in the fifth layer of the primary motor cortex (Rivara et al., 2003), but not in secondary areas, while the supplementary motor area (SMA) is thought to contain a higher proportion of small-sized cells (Wiesendanger et al., 2007). As cell size correlates with conduction velocity (Lee et al., 1986), which in turn has been shown to correlate with action potential duration (Vigneswaran et al., 2011), this suggests that the reliability of cell-type classification using extracellular spike shapes will depend on the brain region under investigation. For example, in the primary motor cortex (M1) of macaque monkeys, large pyramidal tract neurons have been shown to exhibit spikes with short durations that overlap with those reported for interneurons (Vigneswaran et al., 2011). Additionally, there are significant cross-species differences as well, with the primate cerebrum exhibiting a diverse variety of interneuronal classes (Schmitz et al., 2022).

To address some of these limitations, more sophisticated approaches used not only a single feature, such as action potential duration, but a wider range of information through the unsupervised clustering of spike shape waveforms (Trainito et al., 2019; Lee et al., 2021). While these methods succeeded in identifying distinct cell classes based on neural activity, scrupulous assessment of the reliability of extracellular waveform-based cell classification would require a ground truth dataset of recordings from neurons with verified identifies.

A recent study used optogenetic and pharmacological tools to obtain a curated library from the cerebellum, which they then used to train and validate their semisupervised classifier (Beau et al., 2025). The authors subsequently showed that multiple extracellular metrics fed to a deep learning classifier produced results consistent with expert identification of neuron types in the macaque cerebellum (Herzfeld et al., 2025). However, the applicability of the method to brain regions with less well-defined cell types and anatomical layers is unclear. New ground truth datasets are needed to facilitate the investigation of other brain areas.

In this study, we establish a validated spike shape dataset of interneurons and pyramidal neurons from M1 and SMA in the macaque cortex and directly assess the performance of traditional neural classification. We corroborate earlier results showing a correlation between antidromic latency (ADL) and spike duration in M1 and show that the same relationship also holds in SMA. We present a distribution of optogenetically and antidromically verified interneuron and pyramidal neuron spike durations, demonstrating a substantial overlap between the two. To overcome this, we develop and showcase our unsupervised consensus-based clustering method, ACORN. We evaluate its performance on three distinct datasets and show that the data-driven confidence values it provides are informative. Finally, we discuss promising directions for refining electrophysiological neuron classification, in particular the integration of spatial and temporal firing patterns.

## Materials and Methods

Recordings were obtained from six adult female purpose-bred pair-housed macaques (*Macaca mulatta*, BE, BL, WA, YG; *Macaca fascicularis*, GR, PR). All animal procedures were performed in accordance with the Newcastle University Animal Care Committee's regulations. Data from awake monkeys analyzed in this study were obtained incidentally as part of other scientific projects that involved recordings of optogenetic excitation or pyramidal tract stimulation. Acute recordings were obtained as a secondary objective following the conclusion of the main experiments on animals terminally anesthetized for other projects.

Anesthesia was induced using a balanced combination of ketamine (10 mg/kg, i.m.), medetomidine (3 µg/kg, i.m.), and midazolam (0.3 mg/kg, i.m.) and supplanted with sevoflurane during surgical preparation prior to the start of the experiment (0–2% in 100% oxygen). As sevoflurane is known to suppress neuronal activity (Aksenov et al., 2019), we maintained general anesthesia using a combination of alfentanil (24–73 µg/kg/h, i.v.), ketamine (6–15 mg/kg/h, i.v.), and midazolam (0.3–0.4 mg/kg/h, i.v.) during our recordings. Hartmann's solution (5 ml/kg/h, i.v.) was given to prevent dehydration, and methylprednisolone (5.4 mg/kg/h, i.v.) was given to reduce cerebral edema. A tracheotomy was made to allow positive-pressure artificial ventilation. Arterial blood pressure was monitored through a central arterial line inserted via the carotid artery. The bladder was catheterized. The animal's temperature was maintained with the help of a warm air blanket and a thermostatic heat pad. Anesthetic monitoring consisted of pulse oximeter, heart rate, arterial blood pressure, core and peripheral temperature, and end-tidal CO_2_ measurements.

A multicontact recording probe [a SiNAPS 1,024-electrode NHP probe for Monkey BE (Angotzi et al., 2025) and an Atlas 32-contact E32Tri + R-25-S1-L10 NT probe for Monkeys GR and PR] was mounted on a Kopf micromanipulator, angled to penetrate the cortical region of interest normal to the surface, and slowly advanced using a computer-controlled NaN drive. The PT stimulation electrodes (tungsten insulated with parylene, Microprobes for Life Science, part number LF501G with a tip impedance of ∼10 kΩ) were implanted into the medullary pyramidal tract under physiological control (antidromically evoked cortical potentials). The PT search stimuli consisted of two shocks delivered 10 ms apart (intensity <1 mA; charge-balanced biphasic stimuli, 70 µs per phase).

To calculate the waveforms of putative PTNs, we first applied a 60 Hz high-pass FIR digital filter to 30 ms intervals centered around recorded antidromic stimulation timings to remove any residual 50 Hz interference from the signal. Averaging sweeps from the same channels together (Fig. 1*A*), we obtained user-supplied initial starting points for base, trough, and peak timings (Fig. 1*B*), which were subsequently automatically refined to the local extrema within 160 μs windows. We selected the channel with the greatest combined difference in baseline-to-trough and peak-to-trough voltages. For 1,024-contact SiNAPS probes, we averaged together the two channels that showed the strongest responses rather than relying on only a single contact. We interpolated the signal from the selected channel to a rate of 100 kHz using a Modified Akima spline in order to achieve more precise temporal alignment of troughs across the waveforms.

**Figure 1.**
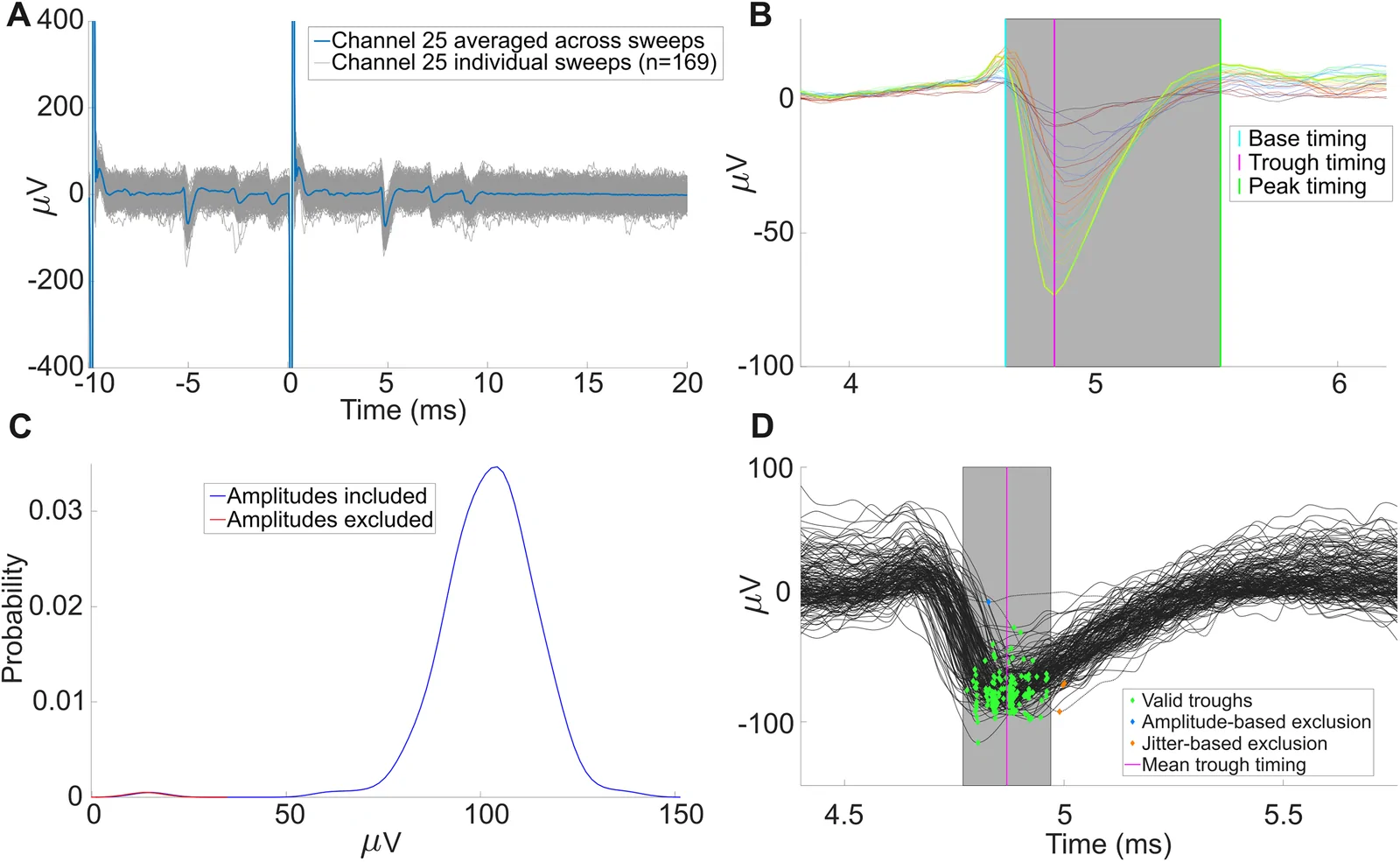
Analysis outline. ***A***, Sweeps are aligned to the timing of antidromic stimulation at *t* = 0. ***B***, The best channel for each spike is selected based on voltage-differences at base, trough, and peak timepoints. These timepoints are automatically refined to the local extrema from user-supplied starting values. ***C***, Spike amplitudes that fall below the left tail of the distribution centered around the primary mode get excluded. The probability density function is estimated adaptively using normal kernels (see Materials and Methods). ***D***, Sweeps with trough timing jitter in excess of 100 µs relative to the expected are excluded. The expected trough timing is the mean of sweeps whose amplitudes were found acceptable in the previous step.

Cells were classified as PTNs when they responded with a consistency of at least 90% across sweeps at a specific ADL and an overall mean absolute amplitude of at least 25 µV. Pyramidal tract neurons are expected to respond consistently to above-threshold PT stimulation, except in rare cases due to collisions with spontaneous spikes. Consistent with this, we found nonresponses to be rare for cells in our PTN dataset, with a median response rate of 97% (Extended Data Fig. 3-1). We considered as nonresponses deflections that fell below the last negative probability change prior to the mode in the kernel density estimated amplitude probability distribution function, across sweeps, of the cell in question (Fig. 1*C*). The bandwidth for kernel smoothing was calculated adaptively per cell using the Improved Sheather–Jones plug-in method (Botev et al., 2010). We also considered as nonresponses spikes with trough timing jitter in excess of 100 µs relative to the mean of the included sweeps (Fig. 1*D*).

Cells were classified as interneurons based on their optogenetic responses. An AAV8-mDlx-Chronos-tdTomato vector (8.06 × 10^12^ vg/ml) was injected at 16 points in M1 over 240 min across 2 d for a total volume of 32 µl (Monkey BL) or injected at 12 points in M1 over 180 min across 2 d for a total volume of 48 µl (Monkeys WA and YG). The mDlx enhancer is reported to be specific for GABAergic interneurons in the forebrain (Dimidschstein et al., 2016), while Chronos is a highly sensitive general use channelrhodopsin with fast kinetics (Klapoetke et al., 2014). Optogenetic responses were first observed after 9 weeks of protein expression.

Concurrent with extracellular recordings using a five-channel Mini Matrix Eckhorn drive (Thomas Recordings), blue light (470 nm) was delivered in short intervals (20–100 ms) at an irradiance of 0.3 mW/mm^2^. Four optical fibers altogether flanked a single recording electrode in an equidistant cross-like arrangement (0.5 mm spacing), with the electrode lying 0.3 mm deeper than the fiber level. To detect and cluster spikes in optogenetic recordings, we used a modified version of WaveClus (Quiroga et al., 2004), in which wavelet-based feature extraction was substituted for UMAP and superparamagnetic clustering for the Leuven method. We then compared firing rates immediately prior to, and during, optogenetic illumination, across intervals of 50 ms, except where optogenetic stimulation lasted shorter than this; then the interval was set to coincide with the duration of the illumination. Where one-tailed Wilcoxon signed-rank tests indicated a significantly higher firing rate during illumination (*p* < 0.05; see Extended Data Fig. 3-1 for exact *p* values) and optogenetic response latency was shorter than 10 ms, we concluded that the cell in question was an interneuron.

Two-millisecond waveform snippets were extracted around detected spikes, with troughs aligned to 0.3 ms into the spike window as recommended by the WaveMAP protocol (Lee et al., 2023). We calculated signal-to-noise ratios (SNRs) for each unit as the mean waveform amplitude divided by twice the standard deviation of the background noise on the channel (Lee et al., 2023) and excluded units with SNRs below 2.7. To normalize the waveforms, we subtracted the median, rather than the mean, of the spike window, because we found the former better-suited to bringing the baseline to 0 µV in the case of large-amplitude spikes. To evaluate the quality of neuron class predictions, we used Adjusted Mutual Information (AMI) as a measure that compensates for chance effects (Vinh et al., 2010): AMI takes on a value of 0 when the mutual information between two clusterings is at chance level and 1 at perfect agreement.

For some of the PTNs, antidromic spike shapes might be distorted by activity synchronous with PT stimuli (early PT volley or other PTNs; Fig. 3*B*). Because this activity is time-locked to the PT stimulus, it will not be averaged out across sweeps. To address this issue, we employed “pattern-matched noise imputation.” In brief, we examined the signal outside the temporal extent of the spike waveforms, and upon finding “breakpoints” that indicated such distortions, we replaced those intervals with noise derived from the same sweep in a way that does not introduce sharp boundaries. A snippet of the code performing this procedure (“cleanspike”) is shared as supplementary material (https://github.com/zolts/ACORN). For consistency, we applied this procedure to all our units: both PTNs and interneurons.

To calculate spike shape characteristics for a unit, we used the waveform obtained by averaging across valid sweeps. We measured the spike width as the period between the trough and the first subsequent datapoint at which the time-derivative of the voltage signal turns negative. We calculated spike amplitudes as the trough voltage relative to the median of the spike window. For PTNs, we measured ADL as the delay between a nerve stimulation event and the start of the consequent negative deflection detected in the cortex (“base timing” in Fig. 1*B*), averaged across valid sweeps.

We developed an unsupervised clustering method, ACORN. We started by dimension-reducing the mean spike waveforms using an optimized form of UMAP (McInnes et al., 2018) that has been shown to improve downstream clustering performance on a variety of datasets: MST-min with Path Neighbors (Dalmia and Sia, 2021). To generate the initial set of constituent clusterings, we employed HDBSCAN (McInnes et al., 2017). We sampled a large region of the parameter space, generating 3*5*5*3*5 = 1,125 individual clusterings, one for each set of sample values (Table 1). We verified that overall results remained robust within this parameter space (Extended Data Fig. 7-1). All other parameters were kept at their default values.

**Table 1.** The region of parameter space used to generate constituent clusterings

Parameter	Sampled parameter values
UMAP n_neighbors	round(N/3, N/4, N/6)
UMAP n_components	4, 8, 16, 32, 64
UMAP min_dist	0.3, 0.4, 0.5, 0.6, 0.7
HDBSCAN min_cluster	12, 16, 20
HDBSCAN min_samples	round(min_cluster / [1.5, 2, 2.5, 3, 4])

Each combination of parameters is used to generate a single clustering; their consensus produces the final result. *N* denotes the number of neurons in the dataset. n_neighbors sets the size of the local neighborhood when learning the manifold structure (a trade-off between interpreting local vs global data structure). n_components sets the number of dimensions the waveform data gets embedded into (a trade-off between the density and the amount of information). min_dist sets the minimum distance between neurons in the low-dimensional embedding (a trade-off between representing local vs global topological structure). min_cluster_size sets the minimum number of neurons that must be present in a group for it to be considered a cluster. min_samples sets the minimum number of neighbors for a neuron to be considered core (a trade-off between exhaustiveness and rigor). We set min_samples as a fraction of the min_cluster_size parameter as shown in the table. Rounding is done using the “half-to-even” tie-breaking rule (IEEE 754), which is the default mode in Python.

We then applied our SEED (Sequential Election of Exemplar Delegates) algorithm to turn multiple HDBSCAN clusterings into a single consensus result. Based on the above, we obtained a matrix of *N* neurons and *M* constituent clusterings. Notably, HDBSCAN does not pre-set the number of clusters it generates, so different constituent clusterings can end up with varying numbers of clusters. Additionally, HDBSCAN can designate potential outliers as “unassigned” instead of necessarily including them in one of the clusters (Fig. 2). SEED is based on the idea that representative members (exemplars) of each final cluster should be similar to members of their own cluster but dissimilar to other exemplars. To identify exemplars, we use the following iterative algorithm (see the code at https://github.com/zolts/ACORN for the exact implementation).

**Figure 2.**
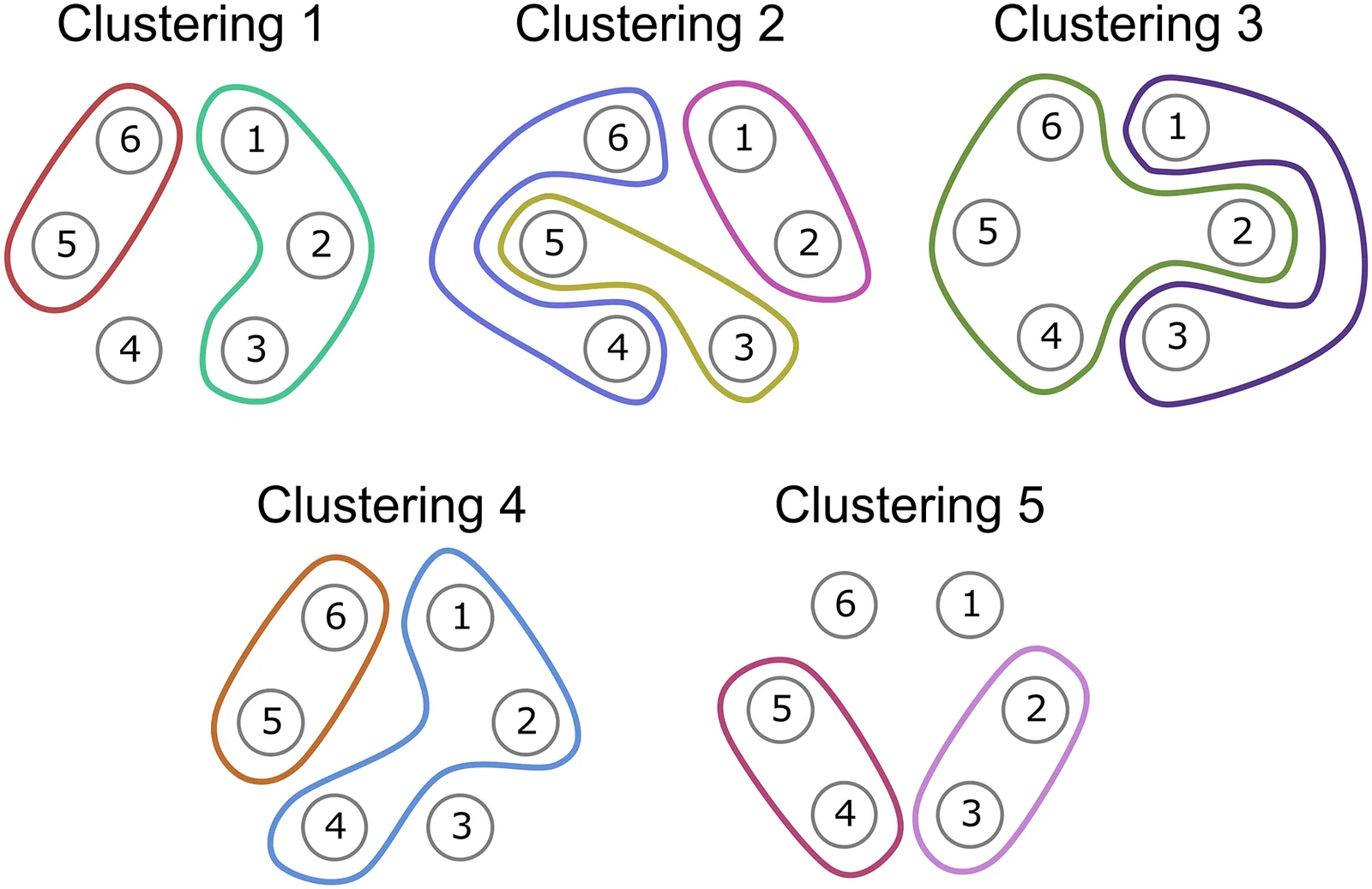
An illustrative example of constituent clusterings in ACORN. Circled numbers represent neurons. Across clusterings, identical neurons are represented with the same number. Colored shapes enclosing sets of neurons represent clusters. Across clusterings, there is no direct relation between clusters; hence, each cluster is represented using a unique color. This arrangement of neurons and clusterings is used throughout Tables 2–5 to illustrate the method using a simple example.

For each neuron *i*, we calculate a “seed metric,” *S_i_*, accumulating information across each clustering *m*. Let *C_i_*_,*m*_ be a cluster into which neuron *i* was assigned in the *m*th constituent clustering. For each neuron *i* and clustering *m*, we calculate *X_i_*_,*m*_, the number of already-identified exemplars that belong to cluster *C_i_*_,*m*_. If *X_i_*_,*m*_ is zero (as by definition would be for the first exemplar), then we increase the value of *S_i_* by the cluster size of *C_i_*_,*m*_ (i.e., number of other neurons assigned to the same cluster as neuron *i* in clustering *m*). This is the similarity part of the metric (Table 2).

**Table 2.** The first exemplar selection iteration on the example dataset

Iteration 1	Neuron 1	Neuron 2	Neuron 3	Neuron 4	Neuron 5	Neuron 6
Clustering 1	3	3	3	0	2	2
Clustering 2	2	2	2	2	2	2
Clustering 3	2	4	2	4	4	4
Clustering 4	3	3	0	3	2	2
Clustering 5	0	2	2	2	2	0
*S* metric (Sum)	10	**14**	9	11	12	10

Numbers correspond to terms of the *S* metric for each neuron as the SEED algorithm identifies exemplars. The positive term is the number of neurons assigned to the same constituent cluster. Neuron 2, achieving the highest *S* value, is chosen as the first exemplar.

If *X_i_*_,*m*_ is greater than zero, then a dissimilarity term (a negative component) is applied to *S_i_*, to ensure that exemplars are distinct from each other. In that case, we subtract from *S_i_* a value of *X_i_*_,*m*_ *N* / *K* / 2, where *K* is the maximum number of clusters in constituent clusterings. We then continue this procedure over each neuron *i* and constituent clustering *m*, adding to and subtracting from *S_i_* as described (Table 3). We thus calculate the *S* metric for each neuron in the dataset. If there are positive *S* values, the neuron with the highest value is chosen as an exemplar. We then recalculate the *S* values for each neuron but with the new exemplar also influencing the results. We repeat this procedure until no positive *S* value is obtained (Table 4).

**Table 3.** The second exemplar selection iteration on the example dataset

Iteration 2	Neuron 1	Neuron 2 (A)	Neuron 3	Neuron 4	Neuron 5	Neuron 6
Clustering 1	−1	−1	−1	0	2	2
Clustering 2	−1	−1	2	2	2	2
Clustering 3	2	−1	2	−1	−1	−1
Clustering 4	−1	−1	0	−1	2	2
Clustering 5	0	−1	−1	2	2	0
*S* metric (Sum)	−1	−5	2	2	**7**	5

Numbers correspond to terms of the *S* metric for each neuron as the SEED algorithm identifies exemplars. The negative term applies instead of the positive term when sharing a constituent cluster with an already-assigned exemplar. The negative term is −N/K/2 = −6/3/2 = −1 in this example, where *N* is the number of neurons and *K* is the maximum number of clusters in constituent clusterings. Neuron 5, achieving the highest *S* value, is chosen as the second exemplar.

**Table 4.** The third exemplar selection iteration on the example dataset

Iteration 3	Neuron 1	Neuron 2 (A)	Neuron 3	Neuron 4	Neuron 5 (B)	Neuron 6
Clustering 1	−1	−1	−1	0	−1	−1
Clustering 2	−1	−1	−1	2	−1	2
Clustering 3	2	−2	2	−2	−2	−2
Clustering 4	−1	−1	0	−1	−1	−1
Clustering 5	0	−1	−1	−1	−1	0
*S* metric (Sum)	−1	−6	−1	−2	−6	−2

Numbers correspond to terms of the *S* metric for each neuron as the SEED algorithm identifies exemplars. The negative term is scaled according to the number of already-assigned exemplars in a neuron's constituent cluster. Thus, in constituent clustering 3, where two exemplars are in the same constituent cluster, the negative term is −2. With no neuron achieving a positive *S* value in this iteration, the exemplar selection stage ends.

These exemplar neurons are the seeds of the final clusters. While constituent clusterings may vary in the number and pattern of cluster labels they assign, the same neurons remain the designated exemplars regardless of the constituent clustering under consideration. Intuitively, the similarity component of the SEED algorithm promotes an arrangement where exemplars are part of large clusters. At the same time, the dissimilarity component encourages each exemplar to belong to a distinct cluster in each constituent clustering. However, in practice, in some constituent clusterings, exemplar neurons may happen to be unclassified or two or more exemplars may sometimes belong to the same cluster.

Each remaining neuron needs to be distributed into one of the final clusters corresponding to one the *L* exemplars or alternatively to be designated as “uncertain” (cluster *L* + 1). We start with *V*, an *N*-by-(*L* *+* *1*) matrix initialized with zeros tallying the votes. For each neuron *i* and each exemplar neuron *k*, we iterate over each constituent clustering *m*. Let *w* be the number of exemplars that belong to the same cluster to which neuron *i* belongs in constituent clustering *m*. If *w* ≥ 1, we increment *V_i_*_,*l*_ by 1/*w*. If *w* = 0, we increment *V_i_*_,*L*+1_ by 0.5. The motivation for the lower increment in the latter case is to ensure that designations of “uncertain” have a lower impact than clashing cluster designations when deriving confidence values from the matrix *V* in the following step.

For each neuron *i*, we then examine its final vote count *V_i_*_,*l*_ across the *L* *+* *1* clusters (Table 5). Neuron *i* is assigned to cluster *l* with the highest *V_i_*_,*l*_ score. If the highest *V_i_*_,*l*_ score is at *l* = *L* + 1, then neuron the classification of the neuron is “uncertain.” The confidence value associated with the assignment is the ratio between the highest and second-highest *V_i_*_,*l*_ scores. Neurons whose assignment confidence values exceed the threshold parameter (2.0 unless otherwise specified) form the overall consensus clustering result.

**Table 5.** Consensus cluster assignments on the example dataset

Voting	Neuron 1	Neuron 2 (A)	Neuron 3	Neuron 4	Neuron 5 (B)	Neuron 6
Clustering 1	A	A	A	Uncertain	B	B
Clustering 2	A	A	B	Abstain	B	Abstain
Clustering 3	Abstain	0.5 for A 0.5 for B	Abstain	0.5 for A 0.5 for B	0.5 for A 0.5 for B	0.5 for A 0.5 for B
Clustering 4	A	A	Uncertain	A	B	B
Clustering 5	Uncertain	A	A	B	B	Uncertain
Exemplar A (Sum)	**3**	**4.5**	**2**	**1.5**	0	0.5
Exemplar B (Sum)	0	0.5	1	**1.5**	**4.5**	**2.5**
Uncertain (Sum/2)	0.5	0	0.5	0.5	0	0.5
Winner	A	A	A	Tie	B	B
Confidence	6.00	9.00	2.00	1.00	9.00	5.00
Consensus	**Cluster A**	**Cluster A**	**Cluster A**	**Uncertain**	**Cluster B**	**Cluster B**

Each neuron, once per constituent clustering, votes for the exemplar with which it shares its cluster. If a neuron shares its constituent cluster with multiple exemplars, its vote is split equally between them. If a neuron's constituent cluster is “unassigned” (−1), it votes for “uncertain” with half strength. If a neuron neither shares its constituent cluster with any exemplars, nor is it unassigned in that constituent cluster, then it abstains. Finally, each neuron whose confidence is above the threshold is assigned to the consensus cluster built around the exemplar that won its votes. If “uncertain” won a neuron's votes or if the neuron's confidence value is less than the threshold, then the consensus result is “uncertain” for that neuron.

### Code accessibility

The ACORN code, as well as an implementation of the “pattern-matched noise imputation” used in this paper, is freely available (https://github.com/zolts/ACORN). The code is not OS-specific. Here, it was run on a Dell Precision 7,920 PC with a Microsoft Windows 10 Enterprise operating system, using Python 3.8 (Python Software Foundation) and MATLAB R2025b (MathWorks).

## Results

To establish ground truths, we identified PTNs based on antidromic stimulation (Fig. 3*A*). Multicontact probes allowed us to distinguish between spikes from spatially separated cells within recordings (Fig. 3*B*). We used the shapes of reliably evoked antidromic spikes (Fig. 3*C*) for neuronal classification. We identified interneurons through optogenetics. We first spike sorted all recorded neurons on a given electrode and then verified the responses of individual clusters to optical stimulation (Fig. 4).

**Figure 3.**
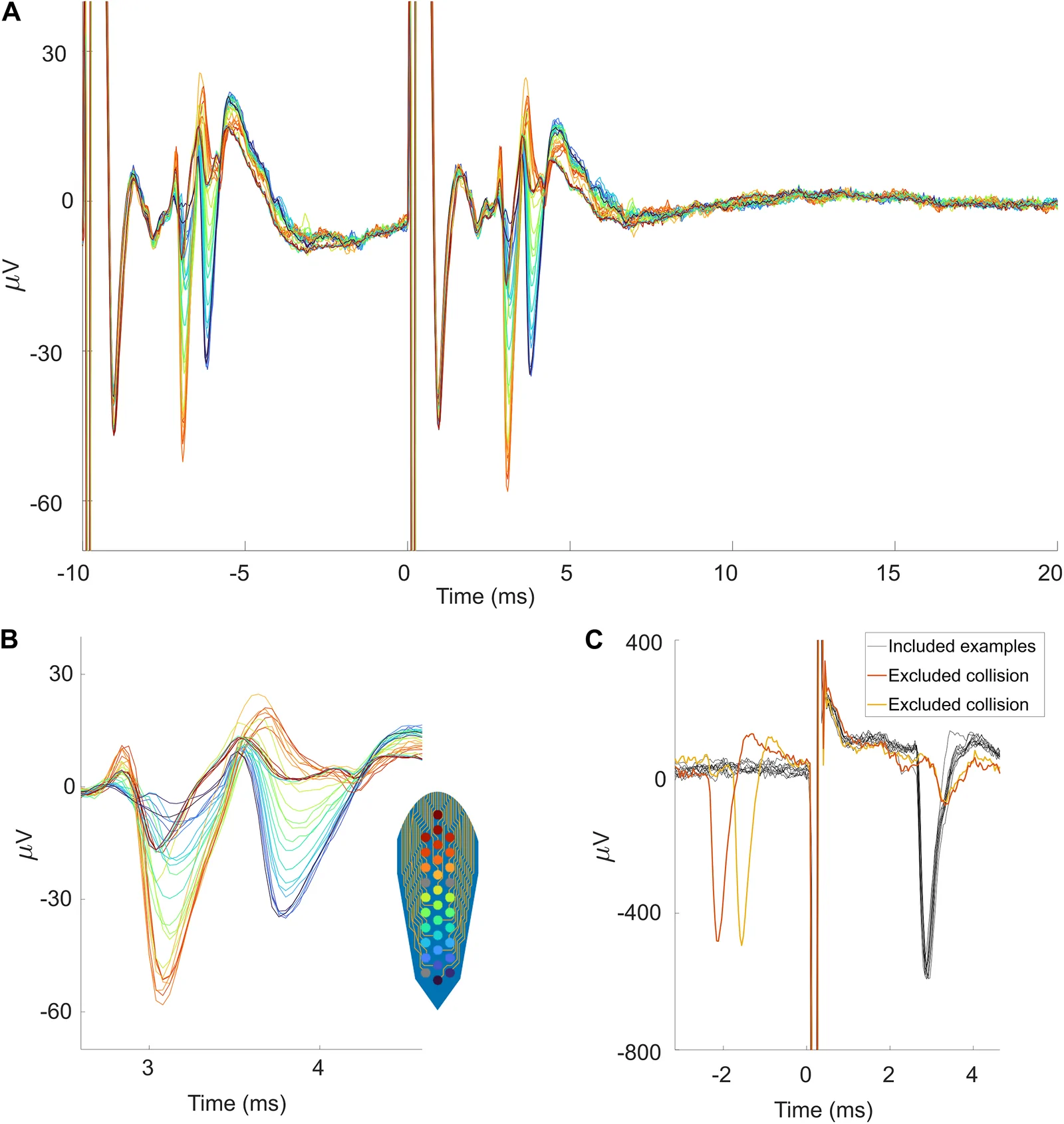
Ground truths for PTNs. ***A***, Dual pulse antidromic stimulation, 10 ms apart, evokes characteristic spikes at defined latencies. Traces are channel-averages of sweeps. ***B***, Spikes with similar timings can be distinguished due to spatial separation across the contacts. The colors of the traces correspond to the physical location of the recording channel as indicated on the inset picture of the electrode tip (based on an image supplied by Atlas Neuro). In this example, the earlier spike (red) localizes to superficial channels, while the later spike (blue) to near the tip of the probe. Gray circles represent contacts from which no data were obtained. ***C***, Spontaneous spikes generated prior to antidromic stimulation can suppress the expected PTN response, as the two action potentials collide along the axon. We provide a dataset with spike waveforms for all identified neurons: 65 PTNs and 22 interneurons (Extended Data Fig. 3-1).

10.1523/ENEURO.0394-25.2026.f3-1 Figure 3-1 Our ground truth dataset. Metadata about each neuron include species, sex, animal ID, state (awake or anesthetized), brain area, probe type, cell type (pyramidal tract neuron or interneuron). Electrophysiological measurements include number of spikes, average spike widths and amplitudes, and signal-to-noise ratios. For PTNs, antidromic latencies and response rates are shown (see Methods for details). For interneurons, optogenetic facilitation denotes the ratio of firing rates prior to vs during intervals of light stimulation with corresponding p-values (see Methods for details). Normalized waveforms are provided for each of the 87 ground truth units at a rate of 100 KHz over 2 ms. Extracellular troughs are aligned at t = 0.3 ms, with the value of the trough normalized to -1 there (see Methods for details). Download Figure 3-1, XLS file.

**Figure 4.**
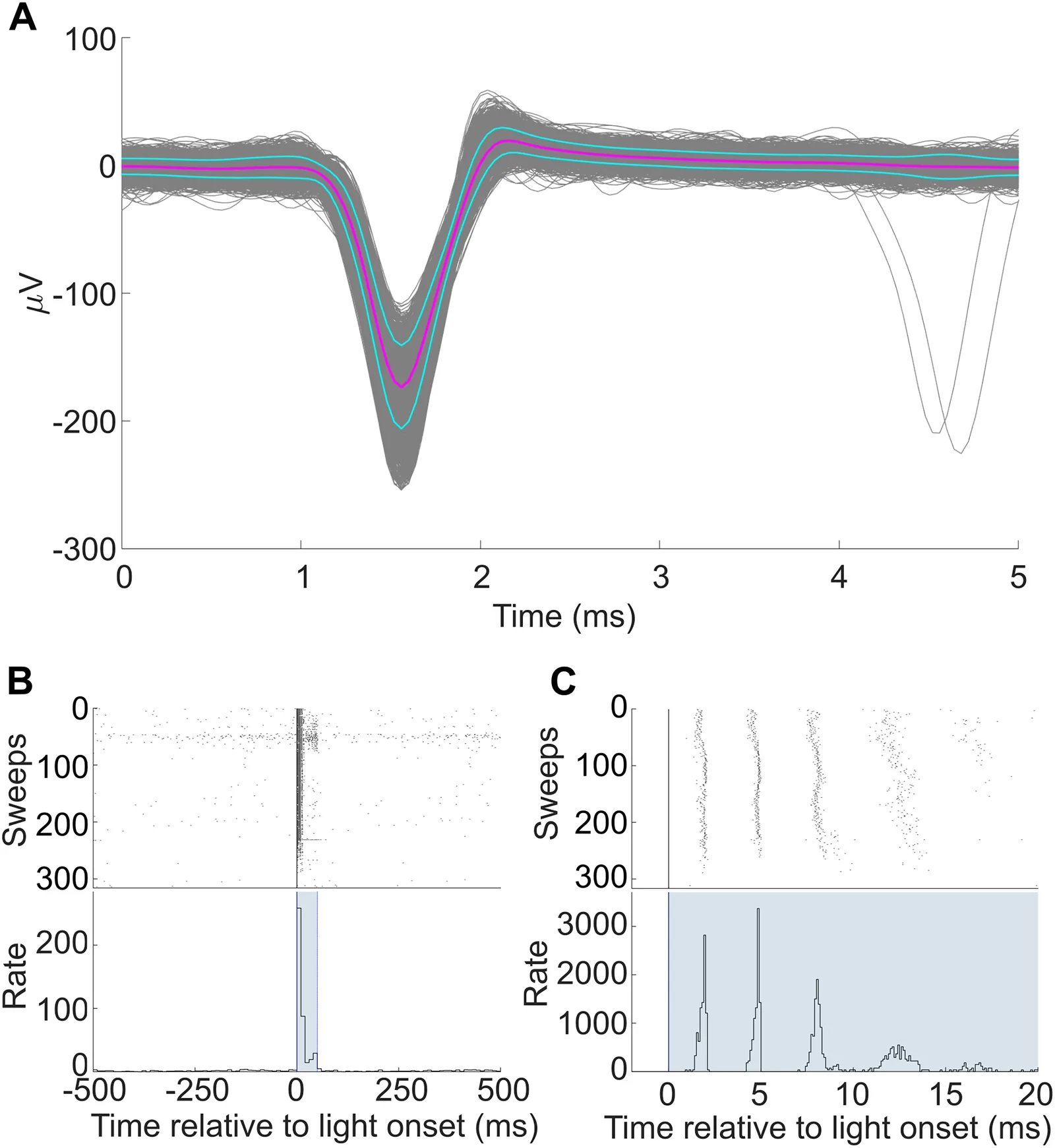
Ground truth for interneurons. ***A***, Individual spike waveforms (gray traces) and their mean and standard deviation (magenta and cyan traces, respectively). ***B*, *C***, Cells are considered interneurons if they respond to optogenetic stimulation (***B***) elicited through a channelrhodopsin driven by an interneuron-specific promoter (see Materials and Methods) with a burst of activity (***C***). We provide a ground truth dataset (Extended Data Fig. 3-1).

We confirmed the finding that spike width correlates with ADL in M1 and found that the same correlation also holds in SMA (Fig. 5). This suggests that trough-to-peak spike width is a physiologically meaningful measure. We observed longer trough-to-peak PTN widths in M1 than some previous works (Vigneswaran et al., 2011). This may be due to differences in recording sites, macaque species, or the effects of general anesthesia, which can increase repolarization times (Jia et al., 2024).

**Figure 5.**
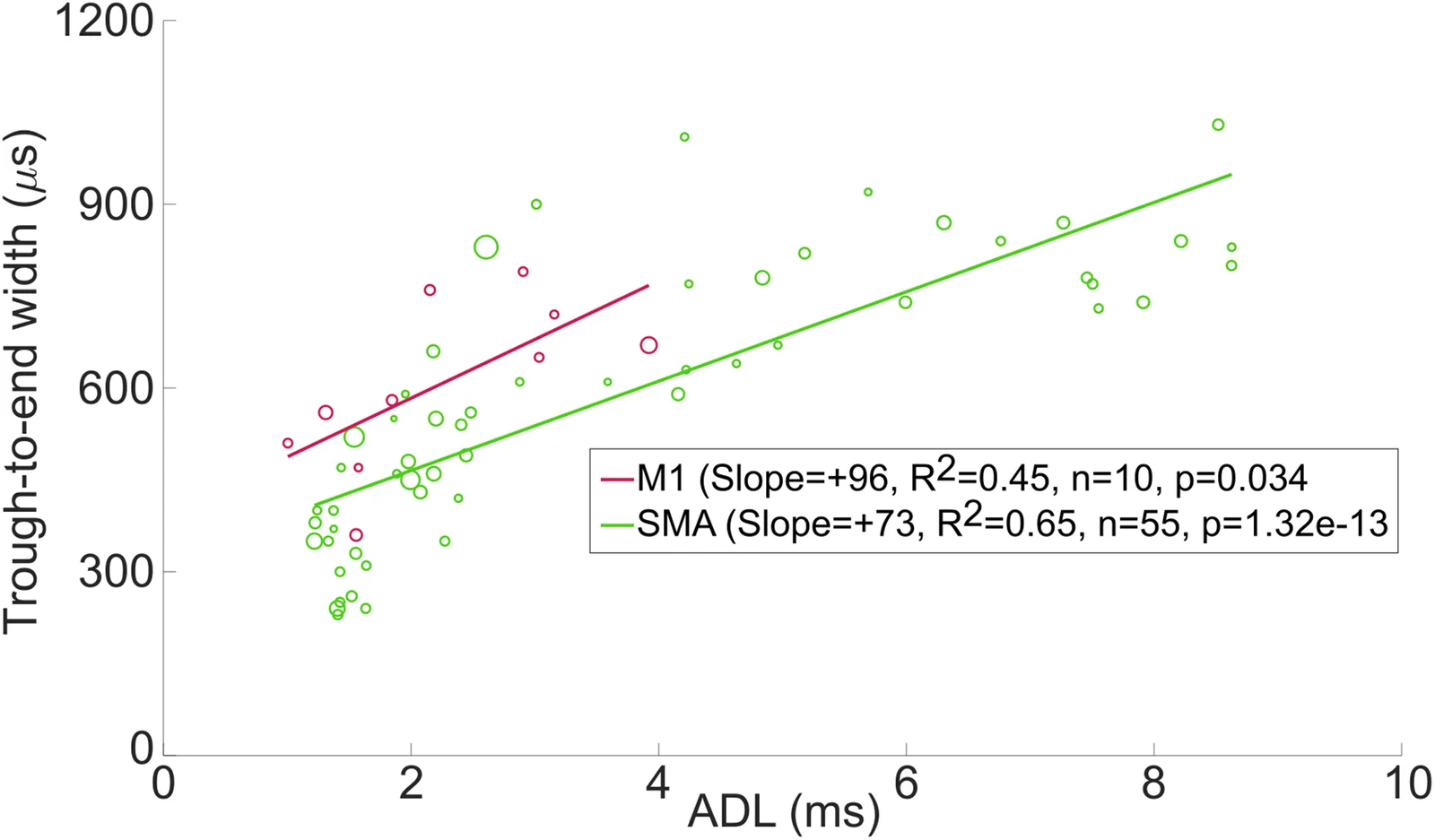
Spike width correlates with ADL in anesthetized animals in both M1 and SMA. Marker diameters relate linearly to spike amplitudes. Linear regression models yielded R^2^ of 0.45 and 0.65 and *p* values of 0.034 and 1.32 × 10^−13^ in M1 and SMA, respectively.

We found a substantial overlap in spike widths between PTNs and interneurons (Fig. 6*A*), raising questions about the reliability of spike width as a distinguishing feature between the two classes. Many previous studies used a spike width value between 0.2 and 0.4 ms as the threshold for discrimination (reviewed in Vigneswaran et al., 2011). Careful choice of spike width threshold could affect classification performance. Hence, to determine how well such an approach would perform on our dataset, we calculated the AMI between predicted and true binary classifications with thresholds covering the width range used in the literature (0.2–0.4 ms). This served as a blind test in the sense that no information about the true identities of the units was used in picking the exact threshold. We observed highly variable performance using the spike width-based approach (AMI = 0.15 ± 0.07 mean ± SD), confirming that threshold choice strongly affects the results. This prompted us to seek a more robust approach to cell-type classification.

**Figure 6.**
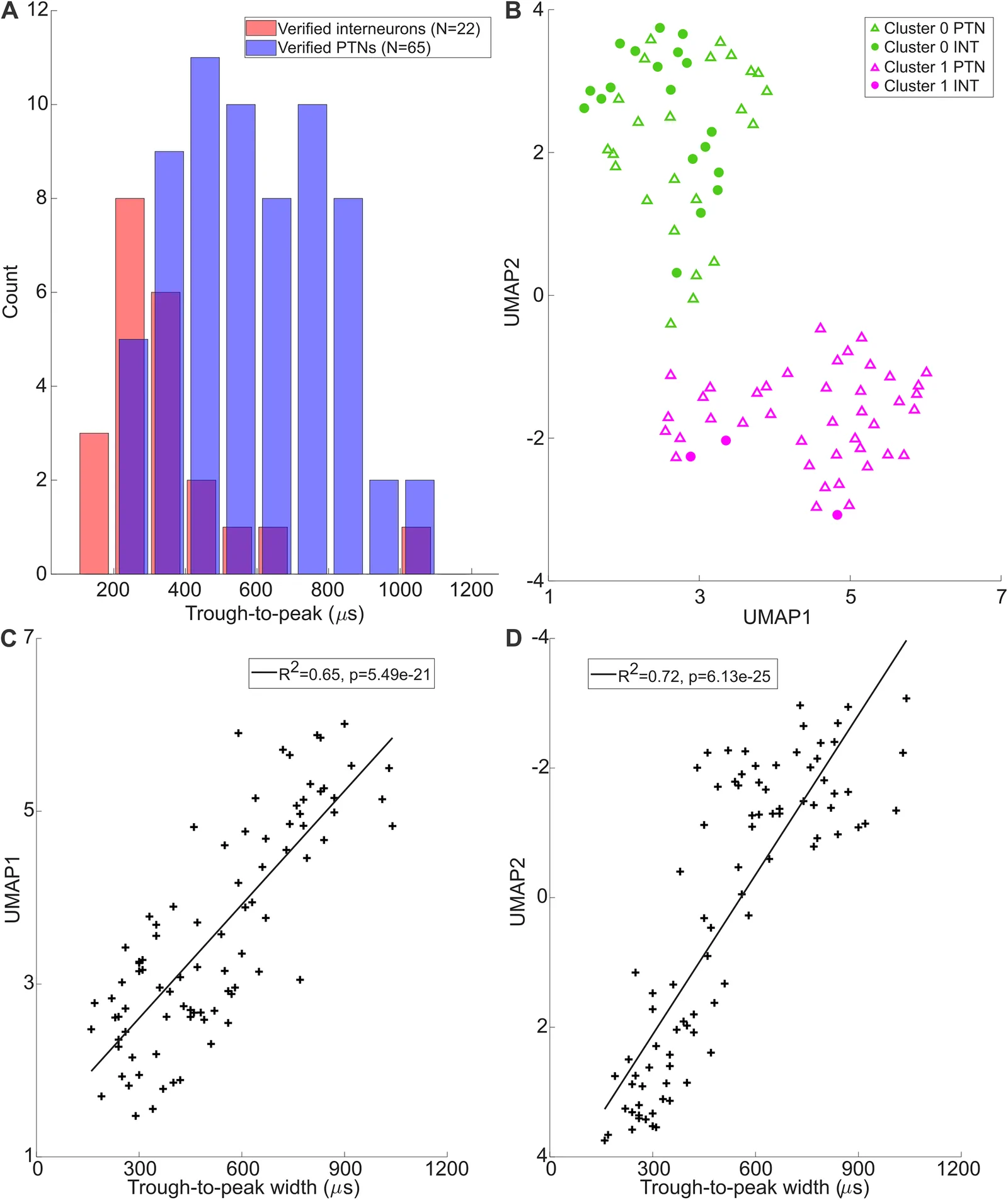
While broad-width PTNs are largely distinguishable from interneurons, narrow-width PTNs and interneurons are not readily separable using width-based approaches. ***A***, Spike widths of PTNs and interneurons overlap substantially, especially ∼300 µs. While on average PTNs have longer action potential durations than interneurons, both short-width PTNs and long-width interneurons exist. ***B***, The WaveMAP unsupervised approach produced two clusters: one enriched for PTNs (Cluster 1) and another that is a roughly equal mixture of the two neuron classes (Cluster 0). ***C*, *D***, Linear regressions indicate that both UMAP dimensions correlate significantly with spike width (*F* tests against constant models: *p* = 5.49 × 10^−21^ and *p* = 6.13 × 10^−25^ for UMAP1 and UMAP2, respectively).

Trough-to-peak duration is a salient feature, but not the only piece of information a spike waveform contains. We therefore asked if a feature-agnostic method could discriminate between these two classes of neurons more accurately than a naive spike width-based approach did. We applied WaveMAP (Lee et al., 2023) to the normalized average waveforms of our units, clustering them using the Louvain method based on UMAP-derived graph representation (Fig. 6*B*). While WaveMAP succeeded in identifying a cluster heavily enriched for PTNs (false discovery rate of only 7% with our dataset), it had low specificity for interneurons, with a false discovery rate of 56% (Table 6). Despite the unbiased nature of the approach, we found a strong correlation between the spike width measure and the dimension-reduced forms of normalized waveforms represented by UMAP values (Fig. 6*C*,*D*).

**Table 6.** Confusion matrix of the WaveMAP method applied to our units

WaveMAP, our dataset (AMI: 0.14)	Actual interneuron	Actual PTN
Cluster 1 (“Interneuron”)	19	24
Cluster 2 (“PTN”)	3	**41**

In each row (cluster), the highest value is underlined. In each column (ground truth class), the highest value is highlighted in bold if it is underlined.

While the unbiased approach taken by WaveMAP showed promise, we identified a number of aspects that could be improved: First, the Louvain method employed has been shown to suffer from a “major defect” (Traag et al., 2019) that can result in arbitrarily badly connected communities. Second, WaveMAP draws hard boundaries, assigning each neuron exhaustively as part of a cluster. This approach may work well with datasets containing distinct and well-represented clusters but could be sensitive to the inclusion of outliers or neurons whose waveforms are ambiguous or intermediate. Third, while it clusters all units, it provides no confidence values for those assignments: archetypal members of a group and best guesses will be equally part of the same cluster.

We aimed to provide a method that would allow researchers to cluster neurons based on their waveforms robustly and reliably, without training or parameter tuning. An approach that produces an overall consensus result that incorporates information from an ensemble of constituent clusterings over the same dataset has several advantages:

1. The consensus may be a better fit than any of its input clusterings, as it can piece together the overall picture from identified “building blocks.” Differences in the input clusterings can be a positive factor in this context, akin to looking at a scene from different angles.
2. Information from clusterings can be integrated robustly even when they contain different numbers of clusters.
3. Confidence in neurons belonging to their assigned cluster can be quantified. This way, a researcher can choose a confidence threshold according to whether minimizing false inclusions or false exclusions is a greater priority in the paradigm.

UMAP and HDBSCAN feature a number of parameters that can affect their results. Rather than picking a particular set of parameter values tuned to our dataset, we employed an unbiased approach: we iterated over a large region of the parameter space, producing a variety of different clusterings. Our method, ACORN (Adaptive Consensus of Repeated Clusterings), was designed to find a robust consensus of these constituent clusterings (see Materials and Methods). We evaluated cluster predictions against the ground truth along two axes: the proportion of neurons labeled and the accuracy of those labels. The results of the ACORN consensus were better than the average constituent clustering along both axes (Fig. 7*A*). The consensus prediction achieved an AMI of 0.26 while labeling 83% of our units (Table 7).

**Figure 7.**
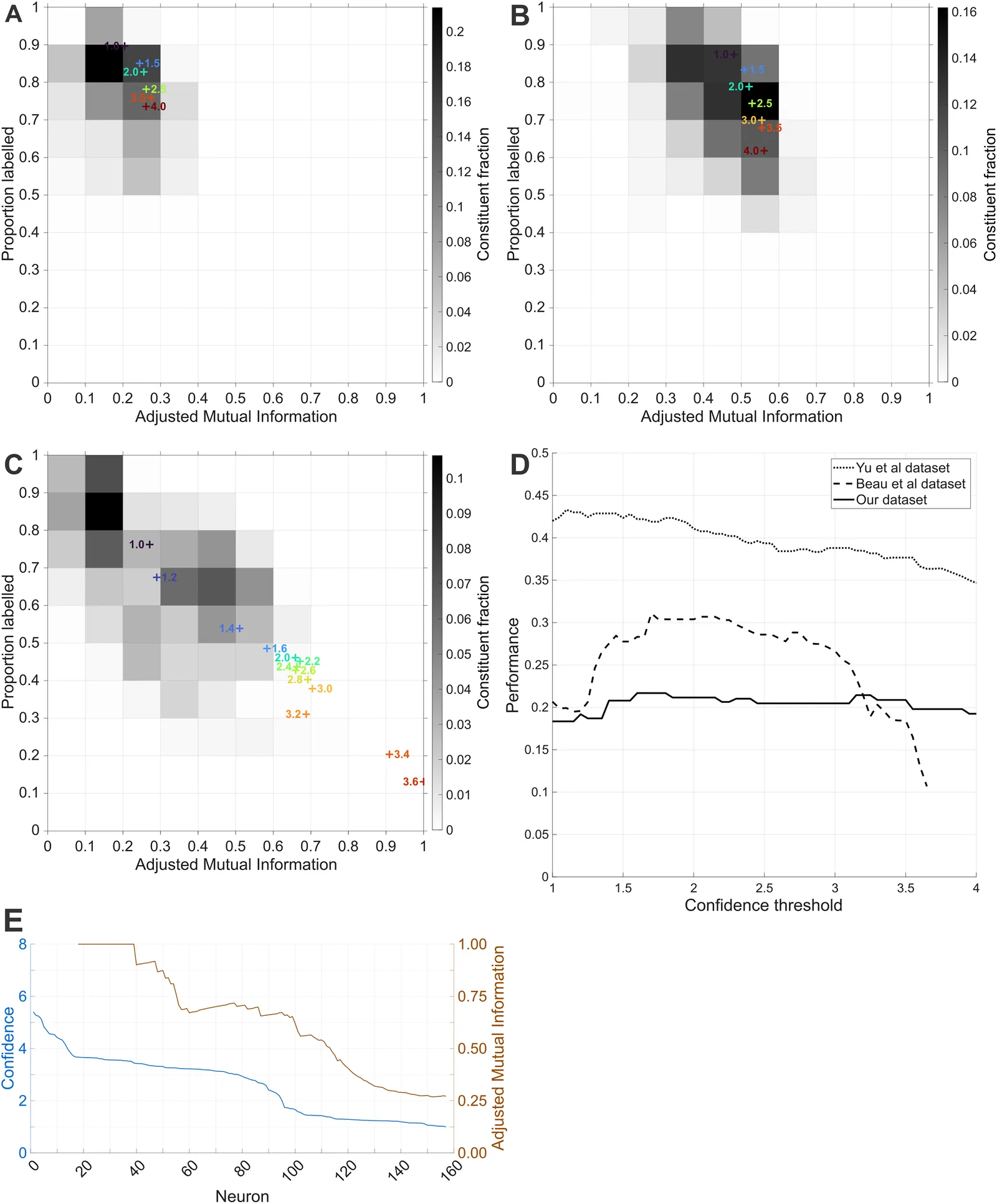
Clustering performance of ACORN evaluated across three datasets. Clustering results for all constituent clusterings (1,125 parameter combinations) are represented in terms of classification performance (AMI) evaluated against the ground truth and the proportion of neurons labeled. The results are shown as a 2D normalized histogram in gray scale. Darker shades represent a higher proportion of constituent clusterings falling within the corresponding ranges along the two axes. Colored crosses with numbers ranging from 1.0 to 4.0 denote consensus clustering results at the specified confidence thresholds. High confidence thresholds produced high AMI at the cost of exhaustiveness, while low confidence thresholds labeled a large proportion of neurons at the cost of AMI. ***A***, ACORN results on our macaque ground truth dataset. ***B***, ACORN results on the Yu et al. dataset. ***C***, ACORN results on the C4 dataset. ***D***, The performance metric (AMI × proportion labeled) plotted as a function of the confidence threshold. As the confidence threshold increases, the proportion of labeled neurons decreases, yet the performance metric remains largely stable within the parameter range of 1.5–3.0 in the three datasets. ***E***, Evaluation of the data-driven confidence metric on the C4 dataset. Including increasingly lower-confidence neuronal assignments in the results produces, on average, worse clustering classification, as measured by AMI. The confidence metric of the assignment of each particular neuron (blue trace) and the AMI result of considering all neurons with at least that much confidence in their clustering assignments (brown trace) are plotted. In this dataset, the 17 highest-confidence assignments were all Purkinje cell complex spikes correctly placed in the same cluster by our algorithm. AMI values are defined where there are at least two distinct neuronal cluster labels to evaluate against the ground truth (*N* > =18). Further analysis of the parameter space is provided in Extended Data Figure 7-1.

10.1523/ENEURO.0394-25.2026.f7-1 Figure 7-1 An exploration of how parameter choice affects clustering results. For each set of parameters (see Table 1), 100 constituent clusterings were generated using different random initial seeds for UMAP initializations and subsequent HDBSCAN clusterings. For each set of parameters, the consensus clusterings were tested against the ground truth on our dataset, characterising clustering performance using Adjusted Mutual Information, proportion of neurons labelled, and a simple product of these two measures (“performance”). Results using different values of the n_neighbors UMAP parameter are shown on different panels: N/3, N/4, and N/6, where N denotes the number of neurons in the dataset. Download Figure 7-1, XLS file.

**Table 7.** Confusion matrix of ACORN applied to our units, using the default confidence threshold of 2.0

ACORN, our dataset (AMI: 0.26)	Actual interneuron	Actual PTN
Cluster 1 (“Interneuron”)	**19**	14
Cluster 2 (“PTN”)	2	**37**
Uncertain	1	14

Cluster predictions are blind; their textual descriptions (in quotation marks within parentheses) are just for convenience to aid interpretation. In each row (cluster), the highest value is underlined. In each column (ground truth class), the highest value is highlighted in bold if it is underlined.

To test the generalizability of our method, we applied ACORN to preprocessed data accompanying the recent PhysMAP paper (Lee et al., 2026). In particular, we used the juxtacellular ground truth dataset recorded from the mouse barrel cortex (Yu et al., 2019). This dataset labels excitatory (E), presumed parvalbumin-expressing (PV^+^), somatostatin-expressing (SOM^+^), and vasoactive intestinal polypeptide-expressing (VIP^+^) interneurons as separate classes. Using renormalized average waveforms, ACORN achieved an AMI of 0.52 while labeling 79% of the neurons (Table 8; Fig. 7*B*). Treating different layers as different classes, ACORN achieved an AMI of 0.36 while labeling the same 79% of neurons (Extended Data Table 8-1).

10.1523/ENEURO.0394-25.2026.t8-1 Table 8-1 Confusion matrix of ACORN applied to the Yu et al dataset, using the default confidence threshold of 2.0, with 8 distinct ground truth classes incorporating layer information. “E-2/3” refers to “excitatory cell in layer 2 or 3”; similarly, a suffix of “-4” indicates layer 4 cells, and “-5” layer 5 cells. Cluster predictions are blind; their textual descriptions (in quotation marks within parentheses) are just for convenience to aid interpretation. In each row (cluster), the highest value is underlined. In each column (ground truth class), the highest value is highlighted in bold if it is underlined. Download Table 8-1, DOCX file.

**Table 8.** Confusion matrix of ACORN applied to the Yu et al. dataset, using the default confidence threshold of 2.0

ACORN, Yu et al. dataset (AMI: 0.52)	Actual excitatory	Actual PV^+^	Actual SOM^+^	Actual VIP^+^
Cluster 1 (“Excitatory”)	**79**	0	7	4
Cluster 2 (“PV^+^”)	0	**34**	0	0
Cluster 3 (“SOM^+^”)	6	21	**42**	1
Uncertain	22	6	20	4

Cluster predictions are blind; their textual descriptions (in quotation marks within parentheses) are just for convenience to aid interpretation. In each row (cluster), the highest value is underlined. In each column (ground truth class), the highest value is highlighted in bold if it is underlined. Here we treated each cell type as its own class, regardless of its layer, given that layer information was not used in our clusterings. For a closer comparison with Lee et al., see Extended Data Tables 8-1 and 8-2, where, still without layer information, ACORN is set to cluster cells types and layers both.

10.1523/ENEURO.0394-25.2026.t8-2 Table 8-2 Confusion matrix of ACORN applied to the Yu et al dataset, using the forced classification paradigm (no confidence threshold), with 8 distinct ground truth classes incorporating layer information. “E-2/3” refers to “excitatory cell in layer 2 or 3”; similarly, a suffix of “-4” indicates layer 4 cells, and “-5” layer 5 cells. Cluster predictions are blind; their textual descriptions (in quotation marks within parentheses) are just for convenience to aid interpretation. In each row (cluster), the highest value is underlined. In each column (ground truth class), the highest value is highlighted in bold if it is underlined. Download Table 8-2, DOCX file.

To compare with PhysMAP, we calculated the Modified Adjusted Rand Indices (MARI) for our clustering results as well. This was 0.38 for the eight-class scenario at the default confidence threshold and 0.31 when ACORN was forced to classify all units regardless of confidence levels (Extended Data Table 8-2). On the same dataset, PhysMAP achieved a MARI of ∼0.32 when using full multimodal information or ∼0.28 when using only the waveforms in an equivalent comparison (Lee et al., 2026, their Fig. 2*C*).

To further test the robustness of our clustering technique, we applied it to data from the recently published C4 database (Beau et al., 2025). This dataset also derives from a different brain area, namely, the cerebellum, and includes ground truth labels of five distinct cell types. By processing the waveform data in the same way and clustering them using the same sets of parameters as before, our method achieved an AMI of 0.66, labeling 46% of the dataset (Table 9; Fig. 7*C*).

**Table 9.** Confusion matrix of ACORN applied to the c4 dataset, using the default confidence threshold of 2.0

ACORN, C4 dataset (AMI: 0.66)	Actual GoC	Actual MFB	Actual MLI	Actual PkCcs	Actual PkCss
Cluster 1 (“MFB”)	0	**25**	1	0	6
Cluster 2 (“PkCcs”)	0	0	0	**20**	0
Cluster 3 (“PkCss”)	0	0	4	1	**38**
Uncertain	18	5	23	38	27

Cluster predictions are blind; their textual descriptions (in quotation marks within parentheses) are just for convenience to aid interpretation. In each row (cluster), the highest value is underlined. In each column (ground truth class), the highest value is highlighted in bold if it is underlined.

Both high AMI and a high proportion of neurons labeled are desirable in clustering results. By adjusting the confidence threshold, it is possible to trade-off the two measures against each other. To investigate this relation, we introduced a “performance” metric defined as a simple product of the AMI and proportion labeled values. Across the three different datasets, we observed that this performance score was stable around our default confidence threshold of 2.0 but declined at extreme values on the C4 dataset (Fig. 7*D*).

To examine the relation between our blindly obtained confidence values and ground truth agreement, we calculated the AMI values sequentially for the *n* neurons whose cluster assignments were most confident in the C4 dataset. We expected a group of high-confidence assignments to produce high AMI and that adding increasingly lower-confidence predictions to the set would gradually reduce the AMI. This is indeed what we observed (Fig. 7*E*). Notably, the 39 highest-confidence assignments achieved a perfect AMI of 1.00 on this dataset. Taken together, these results indicate that our data-driven confidence metric, derived without reference to the ground truth labels, meaningfully relates to clustering performance.

## Discussion

A single parameter, such as spike width, cannot capture the totality of the electrophysiological properties of a cell, so it is unsurprising that some overlap can be observed among different classes of neurons along what is effectively a one-dimensional projection of a complex feature space. What is more remarkable is that we find this overlap in the macaque cortex to be more significant than previously reported, with typical spike width thresholds used in previous works unable to select interneurons reliably.

We observed a notable difference in PTN and interneuron spike width distributions, with a corresponding asymmetry in classification performance. A majority of our PTNs exhibited long spike widths that make them easily distinguishable from interneurons, which tend to have shorter widths. However, the presence of “fast PTNs” made reliable identification of interneurons challenging, as the former can have similarly “thin spikes” as the latter. Here we extend this observation to the SMA, suggesting it as a more general feature of the motor cortex rather than a peculiarity of M1.

While we used an optogenetic approach to target GABAergic interneurons for our ground truth dataset, the group likely contains a mixture of both parvalbumin- (PV) and somatostatin-expressing (SOM) interneurons, which may have different waveform and spiking properties. In particular, a recent study suggests that SOM subtypes can exhibit spike durations ranging from <0.4 ms to over 2 ms (Morales et al., 2021), which challenges the idea that a singular spike width threshold, however well chosen, would allow for neuron subtypes to be reliably distinguished from each other.

What might affect the extent of the overlap observed in different studies? One important consideration is interspecies differences in gene expression and therefore in neuronal ion channel composition. While in rodents the fast potassium channel Kv3.1b is expressed in interneurons but only rarely and weakly in PTNs, in the macaque the same channel is present on both classes of neurons (Soares et al., 2017), resulting in more similar spike widths between the two. Relatedly, there is a large diversity of interneuron subtypes in primates (Krimer et al., 2005). A greater variety of interneuronal subtypes could occupy a more substantial volume in electrophysiological feature space, which could contribute to the greater overlap in PTN and interneuron spike widths observed in primates.

The performance of spike width-based classification heavily depends on the threshold value chosen, typically between 0.2 and 0.4 ms. We characterized it by calculating the average AMI of the range, representing a “typical” performance in the absence of guiding information. Setting this as a bar, we assessed the performance of the unsupervised classification technique WaveMAP on our dataset and found it to be similar, as measured by AMI, as spike width-based classification. Furthermore, low-dimensional UMAP representations of the units strongly correlated with their spike width measures, underscoring that spike width is a salient piece of information represented in the spike waveform snippets in the context of binary classification.

AMI has several desirable properties for comparing clusterings. It corrects for chance effects: the expected AMI of a random clustering compared against the ground truth is zero regardless of the number of classes and neurons. AMI uses an information-theoretical approach that works well even on unbalanced datasets. However, one downside is its nonmetricity: a set of three clusterings can violate the triangle inequality if AMI is used as a distance measure. The MARI also corrects for chance effects but also violates the triangle inequality. The main difference between the two is that MARI is based on pairwise element comparisons, whereas AMI measures overall information overlap.

Normalized Information Similarity (NIS) is a related measure that obeys the triangle inequality; however, NIS violates the constant baseline property: random clusterings will have different expected NIS values against a ground truth dataset depending on the number of clusters. Overall, we consider AMI the most useful measure for assessing different clusterings against the same ground truth dataset—but with the notable caveat that AMI values across different datasets are not numerically comparable.

For any new method aiming to classify cells based on measurements of their electrophysiological properties, a solid ground truth dataset is crucial to serve as its foundation and to validate its reliability. Importantly, such a dataset should contain a good representation of the cell types under investigation, to enable training and assessing suitable classification models. In this study, we exploited antidromic stimulation and optogenetic interventions to establish our validated dataset. Critically, this included both fast and slow PTNs from two cortical different areas, with ADLs ranging from 1.0 to 8.6 ms in the SMA.

With a larger number of units in a dataset, more subtle differences beyond that of spike width may become significant. In particular, the developers of WaveMAP recommend “300 high-quality units” for reliable classification (Lee et al., 2023). Thus, while we used the same 87-unit ground truth dataset as a test bed to compare different classification approaches, WaveMAP may have performed better if supplied with a larger dataset. Another important factor is the SNR cutoff imposed on the dataset: WaveMAP recommends a default value of 3.0 but suggests that the threshold be relaxed when investigating low-amplitude spiking units (Lee et al., 2023). As our recordings included many well isolated but low-amplitude units, we settled on an SNR threshold of 2.7.

Given that reliably ascertaining neuron identities in vivo is a challenging task, and so verified ground truth datasets are likely to remain relatively small in the foreseeable future, we decided to explore steps we could take to improve classification results even with a limited dataset. We introduced “ACORN,” an ensemble clustering technique acting on normalized waveform snippets that uses UMAP for dimension reduction and HDBSCAN for classification. We used the consensus of 1,125 clustering iterations with different sets of parameters to obtain the ultimate classification results. This had the added benefit of providing a confidence measure of each unit's group assignment.

ACORN outperformed both WaveMAP and classical spike width-based approaches on our dataset, as measured by AMI, suggesting that our method effectively incorporated more relevant information than just the spike width measure into the classification decisions. Notably, we did not fine-tune the parameters for our dataset but covered a large region of the parameter space; this enabled our method to perform well out-of-the-box on substantially different datasets (Yu et al., 2019; Beau et al., 2025; Lee et al., 2026).

While in this study we applied ACORN to normalized spike waveforms to cluster neuronal cell types, as is likely to be the most common use case in electrophysiology, the method itself is more generally applicable. There is no requirement that the input data be waveforms nor indeed that they derive from neurons. The consensus step (SEED) of the pipeline is universal and can be applied to any set of constituent clusterings to produce a consensus result with concomitant confidence values. Importantly, the number of final clusters is algorithmically derived from the data rather than from user input, underlining the blind and unbiased nature of the method.

A promising next step in building a more advanced classification method would be to incorporate information not just from the spike waveforms of individual contacts but also from the temporal (Yu et al., 2025; Lee et al., 2026) and spatial characteristics of the units. The former can be effectively investigated by means of “three-dimensional autocorrelograms” (Beau et al., 2025), while the latter through examining multiple contacts across a multielectrode probe. This could provide additional clues not only about the spatial extent of the neuron (Kraskov et al., 2020; Ye et al., 2025) but potentially also about its interactions with the rest of the network. For example, correlations in spike timing patterns could indicate excitatory or inhibitory connections between two units captured simultaneously in the same recording, which, in turn, could help produce a verified ground truth dataset (Ren et al., 2020).

Future neuron classification models should incorporate a wider range of information beyond the traditionally used spike width measures, and their performance should be evaluated using solid ground truth datasets. The database we provide can support future research in two main ways. First, it features two distinct neuronal types: PTNs and interneurons, which any classification algorithm should accordingly separate into different clusters—this can be a way to assess the accuracy of classification techniques. Second, it can supplement new datasets obtained from unidentified neurons with a known ground truth dataset: the identity of the cells in our database can be used to label the clusters obtained by unsupervised classification algorithms.

Our unsupervised ensemble clustering technique, ACORN, can be used to label cell types blindly based on waveforms alone without training or parameter tuning. Notably, it provides data-driven confidence values for each cluster assignment it makes, offering researchers a choice between exhaustiveness and a smaller but cleaner subset labeled with high confidence. Its strong out-of-the-box performance on datasets obtained by different labs using a variety of probes in distinct brain areas showcases its robustness, providing an effective tool for electrophysiological cell classification.

## Synthesis

Reviewing Editor: Arvind Kumar, KTH Royal Institute of Technology Decisions are customarily a result of the Reviewing Editor and the peer reviewers coming together and discussing their recommendations until a consensus is reached. When revisions are invited, a fact-based synthesis statement explaining their decision and outlining what is needed to prepare a revision will be listed below. The following reviewer(s) agreed to reveal their identity: NONE.

Synthesis

Some of the concerns of the two reviewers have been addressed. They still remain reserved about the value of the model but it is clear that the ground truth data that the paper brings along is a valuable resource. As you will read in the comments below there are two main points:

(1) you should make the data available and

(2) you should use other open source data to strengthen the applicability of the method.

Reviewer #1

In this revised version of the paper, the authors have answered some of my concerns. However, I think that there are still some problems with the manuscript. The main concern is still that, since the authors made the choice (and I think this is a good one) to make it method-centered, describing more the ACORN methodology with details and a more robust exploration of the parameter space, we are still a bit frustrated by the limited amount of data used to test the algorithmic pipeline now renamed ACORN. Maybe such a methodological paper could benefit from ground truth recordings with known cell types that could be generated for example via MEArec (https://github.com/SpikeInterface/MEArec/). By doing so, the authors would not be limited to a few cell types and/or number of cells, and this would consolidate the core of the paper, before jumping to an illustration with real data.

Other concerns:

-There could be a figure to make the Methods a bit more visual, especially the description of the S metric for each neuron. Currently, this is a bit hard to picture, and I would suggest making it more graphical.

- Figure 1D. I am not sure this is the best representation, with the empty circles, (especially ping and red, hard to spot). Maybe the amplitude-based exclusion zone should also be displayed in gray, similarly as the jitter-based exclusion to properly visualize both.

- What is the main difference between this ACORN approach and previously published consensus spike sorting, such as https://pubmed.ncbi.nlm.nih.gov/27536990/ ?

- The interpolation at 100Khz is still an open question to me. The answer "We believe it does" does not sound precise enough to me, and I would need to be convinced.

- The code should be on a github repo or with a proper url somewhere in the text.

- From a graphical perspective, I am not sure that having lots of single panel figures is the best. Especially when they look alike. For example, Figure 6,7,8 could be merged into a single one, since they share a common message

Reviewer #2

Open access databases already exist https://www.c4-database.com/ and the contribution of this dataset would be a reason for publication in itself given the non-trivial nature of the experiments. I feel the model is not really advancing the current landscape of cell type classification nor I envision I or others trying the model on my own (unlabelled) data given the fact that other models use a much larger set of features (spike field coherence, autocorrelograms, waveforms etc...)

It is not clear that the data supporting the paper will be available to the community (meaning the waveforms and particularly the raw data voltage traces). If it had been the case the contribution to the field would be substantial, as we could add this dataset as a standard benchmarking to score other models.

I would then recommend the paper for publication with minor revisions. I strongly encourage the authors to do ! (apologies if the data release statement was not included in the manuscript !)

Comparison to another model is welcome (C5). But in the spirit of reproducible benchmarks, the models score could be stated explicitly in a table as is commonplace in ML model comparisons techniques, for each dataset.

Also the comparisons should include "unclassified" as a class and compute the accuracy accordingly when comparing with other models.

At last, how should a user choose the number of final clusters (2 for this dataset and then 5 for cerebellum mouse data) ?

## Author Response

Dear Dr Kumar, Thank you for inviting us to submit a revised version of our manuscript to eNeuro.

We thank the reviewers for their comments and suggestions. In particular, questioning the expected number of final clusters as a parameter motivated us to further generalize our method; ACORN no longer requires such information, enabling it to work fully blindly.

To address the two main points that were highlighted in the synthesis:

1. As stated in our manuscript, our database of the waveforms will be available upon publication. Indeed, normalized waveform data for each of our ground truth neurons was already included alongside our previous submission (previously Table 3-1, now relabeled Figure 3-1).

2. In addition to our own dataset, we have used an open source database (C4) to test our algorithm. Newly in this revision, we further extend our analysis to electrophysiological data derived from Yu et al (2019), showing strong blind performance by ACORN on a different brain area, underlining the general applicability of the method.

One of the reviewers recommended that the code be made available at a suitable online repository. We completely agree. The reason we have not yet published ACORN at our URL is to maintain the double-blind review process required by eNeuro. While our code is enclosed as "Extended Data 1", we would like to ask that a link to a public repository be included in the final version if our manuscript is accepted.

Below, we address the reviewers' comments and suggestions point-by-point:

Comments from Reviewer 1 Comment: we are still a bit frustrated by the limited amount of data used to test the algorithmic pipeline now renamed ACORN. Maybe such a methodological paper could benefit from ground truth recordings with known cell types that could be generated for example via MEArec (https://github.com/SpikeInterface/MEArec/). By doing so, the authors would not be limited to a few cell types and/or number of cells, and this would consolidate the core of the paper, before jumping to an illustration with real data.

Response:

We agree that applying ACORN to more datasets would strengthen the paper. However, the suggested tool, MEArec, appears not very suitable for this purpose. MEArec is designed to embed extracellular action potential templates in a time series and add noise to it to test spike sorters: "recordings are built by convoluting and modulating EAP templates with spike trains and adding noise". But ACORN is not a spike sorter. Its starting point is a set of normalized averaged (across action potentials) waveforms, which is analogous to EAP templates. Thus, MEArec's effects are systematically undone by spike sorting and averaging before the point in the pipeline where cell type clustering occurs.

Instead, we used another ground truth test dataset derived from Yu et al, 2019 (https://www.cell.com/neuron/fulltext/S0896-6273(19)30653-1). They obtained juxtacellular recordings in the mouse barrel cortex, identifying excitatory, fast-spiking, somatostatin-expressing, and vasoactive intestinal polypeptide-expressing interneurons as separate classes. Lee et al, 2026 ( https://www.nature.com/articles/s41467-026-71331-0 ) provided average waveforms on this dataset, which we renormalized and fed to our clustering algorithm.

Notably, this dataset is from a different species (mouse), a different brain area (barrel cortex), different number of cell types (four imbalanced classes), and a different recording technique (juxtacellular action potentials have positive polarities). Despite all these differences, ACORN, without any tuning, achieved an AMI of 0.52 with 79% of the neurons labelled. The PhysMAP manuscript shows a Modified Adjusted Rand Index (MARI) of 0.28 on this dataset when using purely waveform information to predict both cell type and layer (orange trace in Fig 2C in https://www.nature.com/articles/s41467-026-71331-0 ). For comparison, ACORN achieved a MARI of 0.38 labelling 79% of the neurons under the same test conditions, or 0.31 if forced to label them all.

Comment: There could be a figure to make the Methods a bit more visual, especially the description of the S metric for each neuron. Currently, this is a bit hard to picture, and I would suggest making it more graphical.

Response: Thank you for this suggestion. We agree, and have introduced a new figure (Fig. 2) that showcases an illustrative example of this part of the algorithm, including the calculation of the S metric for each neuron.

Comment: Figure 1D. I am not sure this is the best representation, with the empty circles, (especially ping and red, hard to spot). Maybe the amplitude-based exclusion zone should also be displayed in gray, similarly as the jitter-based exclusion to properly visualize both.

Response: We have adjusted this panel to use filled diamonds instead of empty circles, and changed the colors to make the representation more visually distinct. Amplitude-based exclusion is performed on each trace relative to its own baseline, and is shown in red in 1C - it cannot be represented in a straightforward way on panel 1D while maintaining the figure axes.

Comment: What is the main difference between this ACORN approach and previously published consensus spike sorting, such as https://pubmed.ncbi.nlm.nih.gov/27536990/ ? Response: The general idea of using a consensus of multiple clusterings to combine information in an unbiased way was presented in an influential 2002 paper by Strehl and Ghosh (Alexander Strehl and Joydeep Ghosh, "Cluster Ensembles-A Knowledge Reuse Framework for Combining Multiple Partitions", Journal of Machine Learning Research 3 (2002): 583-617). This approach has been used in many papers over the years. The paper referenced by the Reviewer (Fournier et al, 2016) uses a consensus-based approach to spike sort. The main differences between ACORN and Fournier et al are: • Different goals and challenges: Fournier et al clusters spikes into units, while ACORN assigns units into clusters. Waveforms are expected to be the same within a unit, but not necessarily within a cluster, requiring a different approach. • Different feature extraction: Fournier et al is based on Principal Component Analysis, while ACORN uses UMAP, a non-linear, geometrically meaningful dimension reduction technique. • Different constituent clustering: Fournier et al use K-means clustering to generate their constituent results, while ACORN uses HDBSCAN, which dynamically finds the number of clusters, can natively find non-spherical clusters, and is less affected by outliers. • Different consensus algorithms: In brief, Fournier et al start with a similarity matrix based on pairwise co-clustering and perform single-link clustering on its additive inverse as the distance, with constraints on cluster size and an inconsistency coefficient measure. They proceed to iteratively merge undersized clusters while gradually increasing the size threshold below which this occurs. During each such iteration, they recalculate their estimates of spike misclassification rates between clusters, regenerate a hierarchal cluster tree, and cut it at a set misclassification probability threshold, thereby merging closely related "core clusters". The loop is repeated until their final number of clusters is maximized. In contrast, ACORN starts with a similarity matrix based on pairwise co-clustering and identifies "exemplar" neurons that are similar to other neurons while being dissimilar from each other. Each of these neurons forms the core of a final cluster. Each other neuron is assigned to one of these final clusters, or to none of them, based on a confidence threshold relating to relative similarity metrics between the neuron in question and the exemplars.

Comment: The interpolation at 100Khz is still an open question to me. The answer "We believe it does" does not sound precise enough to me, and I would need to be convinced.

Response:

To quantitatively investigate the effect of 100kHz interpolation, we have rerun the entire ACORN analysis pipeline on the C4 dataset under different conditions (figure enclosed below).

A clustering method should ideally achieve high AMI while labelling a high proportion of neurons. Thus, in addition to having these two scores represented as our two axes, we also calculated "performance", a straightforward product of the AMI and proportion labelled values. Observing that this performance metric is robust on three datasets between confidence thresholds of 1.8-2.8 (Figure 7D in the manuscript), here we calculated the means and standard deviations of the performance score across this confidence threshold range.

First, we obtained results using waveforms trough-aligned at the original 30KHz sampling rate without any interpolation (panel A here), which achieved a performance score of 0.23{plus minus}0.02. Second, we interpolated to 100KHz and aligned the waveform throughs at this higher sampling rate before feeding them to ACORN (panel B here). This resulted in a notably improved performance of 0.30{plus minus}0.01. Finally, to investigate the source of this improvement, we performed the alignment at 100KHz as before but then downsampled the aligned waveforms back to 30KHz before passing them to ACORN (panel C here). This produced a performance score of 0.29{plus minus}0.01, showing little difference from the full 100KHz condition. Taking these results together, we conclude that more precise waveform alignment after interpolation is the main cause of improvement from interpolation.

Comment: The code should be on a github repo or with a proper url somewhere in the text.

Response: We thank the reviewer for this suggestion. The reason we have not already done so is that eNeuro has a policy of conducting double-blind reviews - publishing the code at our institute's url, or uploading it to a public repository in our name would have unblinded the reviewers. We agree in principle that including a link to a public repo would be preferable for the final publication - and would like to ask the Editors to allow us to make that small technical change if and when our manuscript is accepted.

Comment: From a graphical perspective, I am not sure that having lots of single panel figures is the best. Especially when they look alike. For example, Figure 6,7,8 could be merged into a single one, since they share a common message Response: We have merged the former Figures 6, 7, and 8 into one (Fig. 7), and added results from the Yu et al dataset to the same figure as well.

Comments from Reviewer 2 Comment: It is not clear that the data supporting the paper will be available to the community (meaning the waveforms and particularly the raw data voltage traces). If it had been the case the contribution to the field would be substantial, as we could add this dataset as a standard benchmarking to score other models. I would then recommend the paper for publication with minor revisions. I strongly encourage the authors to do ! (apologies if the data release statement was not included in the manuscript !) Response:

We thank the reviewer for their comment that the dataset would be a "substantial" contribution to the field, and confirm that the dataset will be available for the community upon publication. This was always our intention; indeed, our previously submitted Table 3-1 (labelled "Our ground truth dataset" in the recent round of review) already included waveforms as time series for each of our 87 units. This table is now presented under the name Figure 3-1 per eNeuro manuscript organization guidelines.

We had tried to emphasize the availability of our ground truth dataset in several places in our manuscript; examples include "Our ground truth data can be used as a reference" in the Abstract, "we provide a database of action potential spike shapes" in the Significance Statement, and "The database we provide can support future research" in the Discussion.

Comment: Open access databases already exist https://www.c4-database.com/ and the contribution of this dataset would be a reason for publication in itself given the non-trivial nature of the experiments. [...] Comparison to another model is welcome (C5). But in the spirit of reproducible benchmarks, the models score could be stated explicitly in a table as is commonplace in ML model comparisons techniques, for each dataset.

Response:

We are unsure what "Comparison to another model [...] (C5)" refers to. Our best guess is that the reviewer had in mind the C4 Database, a dataset on which we tested our clustering algorithm in response to their previous suggestion.

We are providing full confusion matrices on each dataset to enable detailed evaluation of the performance of ACORN (Tables 7, 8, 9; Supplementary Tables 8-1, 8-2).

For clarity, we would like to emphasize that ACORN is a geometric clustering method and not an artificial neural network model. It predicts which neurons fall into same or distinct clusters, without predicting what those clusters are (in contrast to supervised machine learning approaches). Metrics based on mutual information are widely used in evaluating clustering performance. Adjusted Mutual Information (AMI), which we report in our manuscript, controls for random chance effects in a rigorous way (Vinh and Bailey, "Information Theoretic Measures for Clusterings Comparison: Variants, Properties, Normalization and Correction for Chance", Journal of Machine Learning Research, 2010).

Comment: the comparisons should include "unclassified" as a class and compute the accuracy accordingly when comparing with other models.

Response:

We believe that this is not the best way to assess the performance of cell type classification methods. Here we list several reasons in support of our belief. First, none of the ground truth datasets include "unclassified" as a distinct class against which clustering predictions could be assessed. Second, "unclassified neurons" are in effect various outliers; they do not form a single distinct class whose members are closely related to each other. Third, the accuracy measure is sensitive to class prevalence, making it less suitable for evaluating datasets with imbalanced classes like the ones we investigated.

Notably, Beau et al similarly report that their percentage of units classified by their semi-supervised machine learning model decreases as their confidence threshold increases (Fig 5C in Beau et al). This is equivalent to an increase in "unclassified" neurons, which they do not treat as a separate class when assessing accuracy. Rather, most of their confusion matrices include a measure of "above threshold" as a percentage (Figs 5D, 5F, 6C, 6D, 6E, S6A, S6B in Beau et al). This is equivalent to our reported measure of "proportion labelled".

Nevertheless, we took multiple steps to alleviate any issues relating to the assessment of our results. First, we reported Adjusted Mutual Information as our primary measure, which is robust against class imbalance and normalizes random chance level to AMI=0. Second, we provided full confusion matrices for each dataset (Tables 7, 8, 8-1, 8-2, 9) for detailed evaluation of our results. Third, in our manuscript we continually reported "proportion labelled" alongside AMI values, with the understanding that an ideal method should score highly on both axes. To further underline this, we provided an additional "performance" metric that incorporates both aspects.

Finally, to perform a direct comparison unaffected by "unclassified" neurons, we forced our method to cluster all neurons in the Yu et al dataset and compared our results (Table 8-2) with those reported by PhysMAP (Fig 2C in Lee et al, 2026). Despite handicapping our method by having its core confidence-assessment part ablated (i.e. in being forced to cluster all), ACRON performed better (MARI=0.31) than PhysMAP on the waveforms (MARI=0.28), as assessed by the Modified Adjusted Rand Index, which is the measure reported in the PhysMAP paper.

Comment: how should a user choose the number of final clusters (2 for this dataset and then 5 for cerebellum mouse data) ? Response: We thank the reviewer for pointing out this weakness of our previous algorithm. We have adjusted ACORN to no longer require such information, enabling it to work fully blindly, and recalculated our results using this updated version, obtaining similar end results as before. We have revised our manuscript accordingly.

## References

[B1] Aksenov DP, Miller MJ, Dixon CJ, Wyrwicz AM (2019) The effect of sevoflurane and isoflurane anesthesia on single unit and local field potentials. Exp Brain Res 237:1521–1529. 10.1007/s00221-019-05528-9 PMC6526065 30919011

[B2] Angotzi GN, Baker AME, Orban G, Vincenzi M, Ribeiro J, Tenorio V, Berdondini L, Baker SN (2025) High-density, identified cell recordings from motor cortex of awake behaving macaques using 1024-channel SiNAPS probes. bioRxiv 2025.07.22.665434. 10.1109/TBME.2026.3694160 42149759

[B3] Beau M, Herzfeld DJ, Naveros F, Hemelt ME, D’Agostino F, Oostland M, Sánchez-López A, Chung YY, Maibach M, Kyranakis S (2025) A deep learning strategy to identify cell types across species from high-density extracellular recordings. Cell 188:2218–2234. 10.1016/j.cell.2025.01.041 PMC12191827 40023155

[B4] Botev ZI, Grotowski JF, Kroese DP (2010) Kernel density estimation via diffusion. Ann Statist 38:2916–2957. 10.1214/10-AOS799

[B5] Dalmia A, Sia S (2021) Clustering with UMAP: why and how connectivity matters. arXiv preprint arXiv:210805525.

[B6] Dimidschstein J, Chen Q, Tremblay R, Rogers SL, Saldi G-A, Guo L, Xu Q, Liu R, Lu C, Chu J (2016) A viral strategy for targeting and manipulating interneurons across vertebrate species. Nat Neurosci 19:1743–1749. 10.1038/nn.4430 PMC5348112 27798629

[B7] Herzfeld DJ, Hall NJ, Lisberger SG (2025) Strategies to decipher neuron identity from extracellular recordings in the cerebellum of behaving non-human primates. bioRxiv 2025.01.29.634860.

[B8] Jia Q, Duan Y, Liu Y, Liu J, Luo J, Song Y, Xu Z, Zhang K, Shan J, Mo F (2024) High-performance bidirectional microelectrode array for assessing sevoflurane anesthesia effects and in situ electrical stimulation in deep brain regions. ACS Sens 9:2877–2887. 10.1021/acssensors.3c02676 38779969

[B9] Klapoetke NC, Murata Y, Kim SS, Pulver SR, Birdsey-Benson A, Cho YK, Morimoto TK, Chuong AS, Carpenter EJ, Tian Z (2014) Independent optical excitation of distinct neural populations. Nat Methods 11:338–346. 10.1038/nmeth.2836 PMC3943671 24509633

[B10] Kraskov A, Soteropoulos D, Glover I, Lemon R, Baker S (2020) Slowly-conducting pyramidal tract neurons in macaque and rat. Cereb Cortex 30:3403–3418. 10.1093/cercor/bhz318 PMC7197198 32026928

[B11] Krimer LS, Zaitsev AV, Czanner G, Kroner S, González-Burgos G, Povysheva NV, Iyengar S, Barrionuevo G, Lewis DA (2005) Cluster analysis–based physiological classification and morphological properties of inhibitory neurons in layers 2–3 of monkey dorsolateral prefrontal cortex. J Neurophysiol 94:3009–3022. 10.1152/jn.00156.2005 15987765

[B12] Lee EK, Balasubramanian H, Tsolias A, Anakwe SU, Medalla M, Shenoy KV, Chandrasekaran C (2021) Non-linear dimensionality reduction on extracellular waveforms reveals cell type diversity in premotor cortex. eLife 10:e67490. 10.7554/eLife.67490 PMC8452311 34355695

[B13] Lee EK, et al. (2026) A multimodal approach for visualization and identification of electrophysiological cell types in vivo. Nat Commun 17:5241. 10.1038/s41467-026-71331-0 PMC13260831 41986318

[B14] Lee KH, Chung K, Chung JM, Coggeshall RE (1986) Correlation of cell body size, axon size, and signal conduction velocity for individually labelled dorsal root ganglion cells in the cat. J Comp Neurol 243:335–346. 10.1002/cne.902430305 3950079

[B15] Lee K, Carr N, Perliss A, Chandrasekaran C (2023) WaveMAP for identifying putative cell types from in vivo electrophysiology. STAR Protoc. 4:102320. 10.1016/j.xpro.2023.102320 PMC10220268 37220000

[B16] McInnes L, Healy J, Astels S (2017) HDBSCAN: hierarchical density based clustering. J Open Source Softw 2:205. 10.21105/joss.00205

[B17] McInnes L, Healy J, Melville J (2018) Umap: uniform manifold approximation and projection for dimension reduction. arXiv preprint arXiv:180203426.

[B18] Morales C, Morici JF, Espinosa N, Sacson A, Lara-Vasquez A, García-Pérez M, Bekinschtein P, Weisstaub NV, Fuentealba P (2021) Dentate gyrus somatostatin cells are required for contextual discrimination during episodic memory encoding. Cereb Cortex 31:1046–1059. 10.1093/cercor/bhaa273 33026440

[B19] Quiroga RQ, Nadasdy Z, Ben-Shaul Y (2004) Unsupervised spike detection and sorting with wavelets and superparamagnetic clustering. Neural Comput 16:1661–1687. 10.1162/089976604774201631 15228749

[B20] Ren N, Ito S, Hafizi H, Beggs JM, Stevenson IH (2020) Model-based detection of putative synaptic connections from spike recordings with latency and type constraints. J Neurophysiol 124:1588–1604. 10.1152/jn.00066.2020 32937091

[B21] Rivara C, Sherwood CC, Bouras C, Hof PR (2003) Stereologic characterization and spatial distribution patterns of Betz cells in the human primary motor cortex. Anat Rec A Discov Mol Cell Evol Biol 270:137–151. 10.1002/ar.a.10015 12524689

[B22] Schmitz MT, Sandoval K, Chen CP, Mostajo-Radji MA, Seeley WW, Nowakowski TJ, Ye CJ, Paredes MF, Pollen AA (2022) The development and evolution of inhibitory neurons in primate cerebrum. Nature 603:871–877. 10.1038/s41586-022-04510-w PMC8967711 35322231

[B23] Soares D, Goldrick I, Lemon RN, Kraskov A, Greensmith L, Kalmar B (2017) Expression of Kv3. 1b potassium channel is widespread in macaque motor cortex pyramidal cells: a histological comparison between rat and macaque. J Comp Neurol 525:2164–2174. 10.1002/cne.24192 PMC5413836 28213922

[B24] Traag VA, Waltman L, Van Eck NJ (2019) From Louvain to Leiden: guaranteeing well-connected communities. Sci Rep 9:1–12. 10.1038/s41598-019-41695-z PMC6435756 30914743

[B25] Trainito C, von Nicolai C, Miller EK, Siegel M (2019) Extracellular spike waveform dissociates four functionally distinct cell classes in primate cortex. Curr Biol 29:2973–2982. 10.1016/j.cub.2019.07.051 31447374

[B26] Vigneswaran G, Kraskov A, Lemon RN (2011) Large identified pyramidal cells in macaque motor and premotor cortex exhibit “thin spikes”: implications for cell type classification. J Neurosci 31:14235–14242. 10.1523/JNEUROSCI.3142-11.2011 PMC3199219 21976508

[B27] Vinh NX, Epps J, Bailey J (2010) Information theoretic measures for clusterings comparison: variants, properties, normalization and correction for chance. J Mach Learn Res 11:2837–2854.

[B28] Wiesendanger M, Hummelsheim H, Bianchetti M, Chen D, Hyland B, Maier V, Wiesendanger R (2007) Input and output organization of the supplementary motor area. In: *Presented at the Ciba Foundation Symposium 132-Motor Areas of the Cerebral Cortex: Motor Areas of The Cerebral Cortex: Ciba Foundation Symposium 132*, pp 40–62. Wiley Online Library. 10.1002/9780470513545.ch4 3322720

[B29] Ye Z, et al. (2025) Ultra-high-density Neuropixels probes improve detection and identification in neuronal recordings. Neuron 113:3966–3982.e12. 10.1016/j.neuron.2025.08.030 PMC12981004 41033305

[B30] Yu H, et al. (2025) In vivo cell-type and brain region classification via multimodal contrastive learning. bioRxiv 2024.11.05.622159.

[B31] Yu J, Hu H, Agmon A, Svoboda K (2019) Recruitment of GABAergic interneurons in the barrel cortex during active tactile behavior. Neuron 104:412–427.e4. 10.1016/j.neuron.2019.07.027 PMC6813892 31466734

[B32] Zaitsev AV, Povysheva NV, Gonzalez-Burgos G, Rotaru D, Fish KN, Krimer LS, Lewis DA (2009) Interneuron diversity in layers 2–3 of monkey prefrontal cortex. Cereb Cortex 19:1597–1615. 10.1093/cercor/bhn198 PMC2693619 19015370

