# Supplementary material for: ACORN: Adaptive Consensus of Repeated Clusterings Identifies Pyramidal Neurons and Interneurons in the Primate Motor Cortex Based on Extracellular Spike Waveforms
Source: eNeuro. 2026 Oct 23;13(10):ENEURO.0394-25.2026. doi: 10.1523/ENEURO.0394-25.2026 (PMC13713218; doi:10.1523/ENEURO.0394-25.2026)
Supplement: Supplementary file 1 [file eneuro-13-ENEURO.0394-25.2026-s004.docx]

| **ACORN**, Yu et al dataset AMI: 0.36, MARI: 0.38 | Actual E-2/3 | Actual E-4 | Actual E-5 | Actual PV^+^-2/3 | Actual PV^+^-4 | Actual PV^+^-5 | Actual SOM^+^ | Actual VIP^+^ |
| --- | --- | --- | --- | --- | --- | --- | --- | --- |
| Cluster 1 (“E”) | 5 | **49** | 25 | 0 | 0 | 0 | 7 | 4 |
| Cluster 2 (“PV^+^”) | 0 | 0 | 0 | 4 | **25** | 5 | 0 | 0 |
| Cluster 3 (“SOM^+^”) | 0 | 0 | 6 | 2 | 8 | 11 | **42** | 1 |
| Uncertain | 0 | 9 | 13 | 1 | 2 | 3 | 20 | 4 |
